# Abdominal Cocoon Syndrome following Primary Subfertility Management with IVF-ET

**DOI:** 10.31729/jnma.3683

**Published:** 2018-08-31

**Authors:** Asmita Pandey, Chanda Karki, Meena Thapa

**Affiliations:** 1 Department of Obstetrics and Gynaecology, Kathmandu Medical College-Teaching Hospital, Sinamangal, Kathmandu, Nepal

**Keywords:** *abdominal cocoon syndrome*, *embryo transfer*, *In Vitro fertilization*, *ovarian Hyper stimulation syndrome*, *sub fertility*

## Abstract

Abdominal cocoon syndrome is a rarely encountered surgical emergency first described by Foo et al. in 1978. This condition is characterised by a thick fibrous membrane which encases the small bowel partially or completely. Diagnosis of this condition is usually made per operatively and the treatment of choice is surgical release of entrapped bowel.

This is a case report of abdominal cocoon syndrome diagnosed during laparotomy done with the provisional diagnosis of abdominal pregnancy following In vitro fertilization- embryo transfer. A 30 Years lady was admitted at Kathmandu Medical College for suspected ovarian hyper stimulation syndrome following In vitro fertilization- embryo transfer. Conservative treatment was done as the first line of management. Failing this, she was treated surgically with the provisional diagnosis of abdominal pregnancy. Abdominal cocoon syndrome was observed intraoperatively. Patient was managed medically with injection due to raised ßhCG level and empty uterine cavity. Intrauterine gestational sac was seen after about seven weeks of In vitro fertilization- embryo transfer. Pregnancy was terminated medically and patient was discharged.

Sub fertility is a common gynecological problem. Its management may sometimes produce challenging health hazards. Thorough screening for medical and surgical illness is very important before proceeding to any kind of assisted reproductive technologies. A multidisciplinary approach is very important to manage such cases.

## INTRODUCTION

Abdominal cocoon syndrome is a rarely encountered surgical emergency first described by Foo et al. in 1978.^1^ Bowel obstruction is a common surgical emergency. However, at times, unusual cases of bowel obstruction such as abdominal cocoon (AC) may be encountered. In this condition, the small bowel is partially or totally encased by a thick fibrocollagenous membrane which gives a cocoon-like appearance. Its clinical features are nonspecific; therefore, AC is often diagnosed intraoperatively.^2^ Development of such syndrome while treating sub fertility can be a real challenge for obstetricians. Infertility is the commonest presentation of genital TB with reported incidence being 40–80% and average incidence of genital TB in infertility clinics world wide is 5–10 %.^3^ AC might lead to tubal infertility and IVF-ET would be the most effective remedy for the patients desiring pregnancy.^4,5^ Here we report a rare case of abdominal cocoon syndrome diagnosed during laparotomy done with the provisional diagnosis of abdominal pregnancy following IVT-ET in a case of primary subfertility, trying to conceive for 10 years with features of acute mechanical intestinal obstruction.

## CASE REPORT

A 30 years lady, referred case brought to Emergency room of Kathmandu Medical College with complaints of abdominal distension after 23 days of IVF-ET.

She is married for 10 years and was trying to conceive for the same duration. She had visited many infertility clinics during this period. Many cycles of ovulation induction was tried on her by different service providers. She has a history of undergoing laparotomy for tuboplasty 4 years back for bilateral tubal obstruction. She has past history of abdominal tuberculosis, managed medically 10 years back.

She underwent IVF-ET and during her follow up in the sub fertility centre, it was observed that even after 22 days of the procedure, beta hCG had failed to rise in blood. Trans-vaginal Sonography (TVS) failed to show the gestational sac. Patient developed painful abdominal distension after about two weeks of IVF-ET. She was admitted in another centre and managed in the line of OHSS. After few days she was referred to our centre along with pigtail in abdomen.

No history of urinary symptoms, breathing problem or electrolyte imbalance was found. Features suggestive of ovarian torsion was absent.

On Clinical examination, patient looked anxious with abdominal and respiratory discomfort. Other vitals were stable except for tachycardia. She had tense and tender abdomen with marked ascites. Fluid thrill test was positive. Her hematological investigations were found normal with serum beta hCG of 92 IU/ml after 3 weeks of procedure. Hence she was admitted with provisional diagnosis of ovarian hyperstimulation syndrome (OHSS). As beta hCG level was slightly higher than the normal, probability of abdominal pregnancy was also kept in mind.

**Figure 1.**
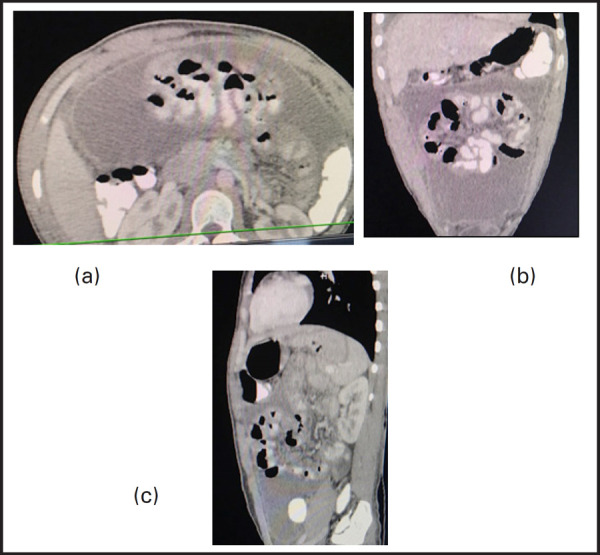
(a) Axial CECT abdomen (soft tissue window) imaging show loculatedascites (solid black arrow) with enhancement and thickening of peritoneum (solid white arrow). (b) Coronal and (c) Sagittal CECT abdomen and pelvis (soft tissue window) imaging show ascites with centrally displaced bowel loops (solid red arrow)._

Contrast Enhanced Computed Tomography (CECT) of abdomen and pelvis Image revealed a large loculated ascites measuring 21x10x19 cm with marked enhancement and thickening of peritoneum, displacing bowel loops posteriorly. Associated omental thickening and matted bowel loops noted. Another 6.1x4.8x5.7 cm loculated fluid collection was found in pouch of douglas (Figure 1).

**Figure 2.**
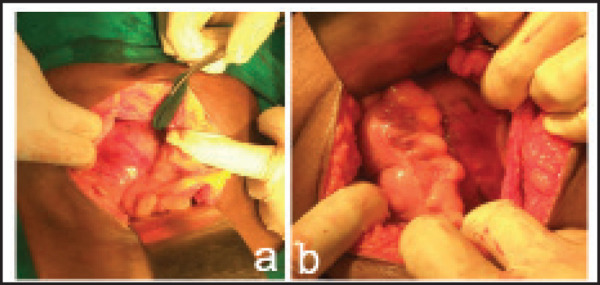
Both (a,b an encapsulated mass containing bowel loops and omentum occupying pouch of douglas with thickened peritoneum.

Conservative management was being done in a high care unit. From the fourth day of admission her abdominal Discomfort and distension increased with loss of bowel sound and increasing tenderness and dehydration with signs of toxicity. Laparotomy wasdone in consultation with the general surgeon on 5^th^ day of admission. A diagnosis of abdominal cocoon syndrome was made during laparotomy (Figure 2). None of the abdominal organs could be visualized or inspected separately. Laparotomy with peritoneal toileting was done.

On Laparotomy, there was 300–400 ml straw colored serosanguinous peritoneal fluid in the peritoneal cavity. Bowel and omentum were encapsulated in the pouch of douglas and supra colic compartment with dense adhesions. Parietal peritoneum was thickened. Peritoneal lavage was done. Post operative period: Uneventful and discharged on 8^th^ postoperative day.

Serum beta hCG was one of the parameter being closely observed. It was going gradually high from 5 IU/ml to 57 IU/ml to 551 IU/ml over a period of three weeks. Transvaginal sonography showed empty uterine cavity. After 33 days of IVF-ET Inj. Methotraxate 50 mg/m^2^ IM was given with the differential diagnosis of pregnancy of unknown location.

Serum hCG continued to rise for another week even after laparotomy and IM Methotraxate. The level reached to 2492 IU/ml. TVS was repeated which revealed an Intrauterine gestational sac of 5 weeks without embryonic pole and cardiac activity after about 40 days of IVF-ET. Medical Termination of intrauterine pregnancy was done and Patient was discharged after 22 days of admission.

## DISCUSSION

AC with infertility belongs to tubal infertility; cause might be encapsulation of fallopian tube and ovaries within fibrous membranes, which limits the activity of fallopian tube and its function of picking up eggs or conveying gametes. As far as infertility treatment is concerned, bowel stimulation and damage should be avoided as much as possible when the diagnosis is confirmed during the operation because it might aggravate the illness and finally give rise to postoperative intestinal obstruction.^5^ Like any other procedures and treatments IVF-ET also have some complications. OHSS is a rare, iatrogenic complication for ovarian stimulation by assisted reproductive technology and other infertility treatments.

In our case patient presented with unique signs and symptoms after IVF-ET. For this patient decision of laparotomy was difficult at the cross road of diagnosing and managing OHSS conservatively versus managing extra uterine pregnancy surgically. Laparotomy was done due to unstable vitals and increasing symptoms of the patient and was diagnosed to have AC. Even after that ßhCG continued to rise in absence of intrauterine gestation so probable diagnosis of pregnancy of unknown location was made. Among the greatest advances in the management of ectopic pregnancy has been the development of medical management, which became available in the mid-1980s.^6^ Therefore, Inj. Methotrexate was given. Medical termination of pregnancy was done to avoid the teratogenicity.

In spite of multiple challenges during diagnosing and treating her, recovery was made possible. However regret is definitely there for not been able to save her precious pregnancy.

## References

[1] Foo KT, Ng KC, Rauff A, Foong WC, Sinniah R. (1978). Unusual small intestinal obstruction in adolescent girls: The abdominal cocoon. Br J Surg..

[2] Hur J, Kim KW, Park MS, Yu JS. (2004). Abdominal cocoon: Preoperative diagnostic clues from radiologic imaging with pathologic correlation. AJR Am J Roentgenol..

[3] Schaefer G. (1976). Female genital tuberculosis. Clin Obstet. Gynaecol.

[4] Hu D, Wang R, Xiong T, Wang ZH. (2013). Successful delivery after IVF-ET in an abdominal cocoon patient: Case report and literature review. Int J Clin Exp Pathol..

[5] Oehninger S, Kreiner D, Bass MJ, Rosenwaks Z. (1988). Abdominal pregnancy after in vitro fertilization and embryo transfer. Obstet Gynecol..

[6] Yip FW, Lee SH. (1992). The abdominal cocoon. Aust N Z J Surg..

